# Gene-Gene and Gene-Environmental Interactions of Childhood Asthma: A Multifactor Dimension Reduction Approach

**DOI:** 10.1371/journal.pone.0030694

**Published:** 2012-02-15

**Authors:** Ming-Wei Su, Kuan-Yen Tung, Pi-Hui Liang, Ching-Hui Tsai, Nai-Wei Kuo, Yungling Leo Lee

**Affiliations:** 1 Institute of Epidemiology and Preventive Medicine, College of Public Health, National Taiwan University, Taipei, Taiwan; 2 Research Center for Genes, Environment and Human Health, College of Public Health, National Taiwan University, Taipei, Taiwan; 3 School of Pharmacy, College of Medicine, National Taiwan University, Taipei, Taiwan; Dartmouth College, United States of America

## Abstract

**Background:**

The importance of gene-gene and gene-environment interactions on asthma is well documented in literature, but a systematic analysis on the interaction between various genetic and environmental factors is still lacking.

**Methodology/Principal Findings:**

We conducted a population-based, case-control study comprised of seventh-grade children from 14 Taiwanese communities. A total of 235 asthmatic cases and 1,310 non-asthmatic controls were selected for DNA collection and genotyping. We examined the gene-gene and gene-environment interactions between 17 single-nucleotide polymorphisms in antioxidative, inflammatory and obesity-related genes, and childhood asthma. Environmental exposures and disease status were obtained from parental questionnaires. The model-free and non-parametrical multifactor dimensionality reduction (MDR) method was used for the analysis. A three-way gene-gene interaction was elucidated between the gene coding glutathione S-transferase P (*GSTP1*), the gene coding interleukin-4 receptor alpha chain (*IL4Ra*) and the gene coding insulin induced gene 2 (*INSIG2*) on the risk of lifetime asthma. The testing-balanced accuracy on asthma was 57.83% with a cross-validation consistency of 10 out of 10. The interaction of preterm birth and indoor dampness had the highest training-balanced accuracy at 59.09%. Indoor dampness also interacted with many genes, including *IL13*, beta-2 adrenergic receptor (*ADRB2*), signal transducer and activator of transcription 6 (*STAT6*). We also used likelihood ratio tests for interaction and chi-square tests to validate our results and all tests showed statistical significance.

**Conclusions/Significance:**

The results of this study suggest that *GSTP1*, *INSIG2* and *IL4Ra* may influence the lifetime asthma susceptibility through gene-gene interactions in schoolchildren. Home dampness combined with each one of the genes *STAT6*, *IL13* and *ADRB2* could raise the asthma risk.

## Introduction

Asthma is the most common allergic disease giving rise to the morbidity or school absence in children [1], [2]. The prevalence of childhood asthma is socially burdensome and results in significant medical expenditure around the world [3]. Many gene and environmental factors are associated with this complex disease, but the effect of each of these factors is mild. It was known that common diseases have complex etiologies such as the dependence of genotypic effects on environmental factors (i.e., gene-environment interactions) and genotypes at other loci (i.e., gene-gene interactions). Recently, there has been increased interest in gene-gene and gene-environment interactions, which may affect asthma pathophysiology.

Inflammatory lung diseases such as asthma [4] are associated with reactive oxygen species (ROS). ROS are regulated by some antioxidant genes and transcription factors. The epoxide hydroxylase (*EPHX1*) and glutathione S-transferase (*GST*) genetic variants are associated with an increased risk for lifetime asthma [5]. Obesity is an important risk factor in asthma [6], [7], [8]. Genetic variations in the obesity-related genes beta-2 adrenergic receptor (*ADRB2*), beta-3 adrenergic receptor (*ADRB3*), insulin induced gene 2 (*INSIG2*), and peroxisome proliferator-activated receptor gamma (*PPARγ*) are also included in our study [9]. Mindful of the importance of inflammation in asthma, we further included several inflammatory genes in our analysis. Interleukin (IL)-13, *IL-4*, interleukin 4 receptor *(IL-4Ra)*, signal transducer and activator of transcription 6 (*STAT6*) and tumor necrosis factor-alpha (*TNFα*) genes are key inflammatory genes in the development of allergic diseases such as asthma [10], [11], [12], [13], [14], [15]. Asthma candidate genes are thought to contribute only 40–60% overall risk [16]. Gene-gene and gene-environmental interactions could explain the residual influence for asthma etiology when a single candidate gene is considered. This study is the first to systematically investigate the potential gene-gene and gene-environmental interactions on various physiological pathway genes on asthma.

Indoor exposure to dampness is suspected to be an important environmental factor for the development of asthma and allergic disease in modern societies. The Taiwan Children Health Study (TCHS) is a population-based study from 14 Taiwanese communities representing a wide range of exposures among school-aged children. This study offers an opportunity to investigate the interactive effects of gene-gene and gene-environmental influences on children's health. The multifactor dimensionality reduction (MDR) approach allows high-dimensional interactions of multiple factors to be simultaneously retrieved, and has successfully identified gene-gene interactions in a variety of diseases including breast cancer [17], essential hypertension [18], type II diabetes [19], atrial fibrillation [20], coronary artery calcification [21], and amyloid polyneuropathy [22]. In the present study, gene-gene interactions for childhood asthma were examined based on 17 SNPs in thirteen candidate genes, encompassing three physiological groups. A number of environmental factors thought to affect asthma were considered, and the associations between candidate genes and environmental factors were explored.

## Results

### Subjects and demographic data

Table 1 shows demographic characteristics and pulmonary function indices for study participants. FEV1, MMEF and FEV1/FVC in asthmatic children are generally lower than non-asthmatic controls. A total of 17 SNPs from thirteen candidate genes were selected for their association with childhood asthma (Table 2). All SNPs were under the Hardy-Weinberg equilibrium (HWE) [23]. Environmental factors including *in utero* smoking, environmental tobacco smoke (ETS), pets at home, incense burning, carpet use, cockroaches in the home and indoor dampness were used to explore the gene-environment interactions. The genotyping call rate for each SNP was over 98% in our study. Data from 1,310 samples were subjected to further gene-gene and gene-environment interaction analysis.

**Table 1:** demographic characteristics and pulmonary function indices for study participants.

	Asthmatics	Controls
Characteristic	(N = 235)	(N = 1,075)
Gender		
Boy	128 (54.5)	522(48.6)
Girl	107 (45.5)	553(51.4)
Age, yr	12.7±1.0	12.8±0.4
BMI	20.8±4.3	20.4±4.1
Pulmonary function indices		
FVC (% predicted)	99.9±13.3	100.1±13.9
FEV_1_ (% predicted)	97.6±14.1	99.9±21.9
MMEF (% predicted)	93.7±24.5	99.7±22.5
FEV_1_/FVC (%)	88.8±6.2	90.9±5.4

Values were presented by n (%) or mean ± SD.

**Table 2:** Genotype characteristics of each single nucleotide polymorphism.

Gene	Chromosome position	Location	Polymorphism	MAF	HWE	*P*-value*
Inflammation SNPs						
*TNFα*, rs1800629	6p21.3	5′ near gene	A/G	0.116	0.135	0.71
*IL4*, rs2243250	5q31.1	5′ near gene	C/T	0.179	1.839	0.18
*IL13*, rs20541	5q31	Exon	C/T	0.313	1.759	0.18
*IL13*, rs848	5q31	UTR-3	G/T	0.324	0.095	0.76
*IL13*, rs1800925	5q31	5′ promoter region	C/T	0.142	4.571	0.03
*IL4Ra*, rs1805010	16p12.1-p11.2	Missense	G/A	0.493	8.216	0.00
*STAT6*, rs324011	12q13	Intron	C/T	0.244	0.064	0.80
Obesity-related SNPs						
*INSIG2*, rs7566605	2q14.2	Intron	C/G	0.399	0.001	0.98
*PPARγ*, rs1801282	3p25	Exon	C/G	0.045	2.391	0.12
*ADRB2*, rs1042713	5q31-q32	Missense	A/G	0.415	2.887	0.09
*ADRB2*, rs1042714	5q31-q32	Missense	C/G	0.096	0.398	0.53
*ADRB3*, rs4994	8p12	Missense	C/T	0.153	0.570	0.45
Antioxidative SNPs						
*EPHX1* exon 4, rs2234922	1q42.1	Exon	A/G	0.137	4.828	0.03
*EPHX1* exon 3, rs1051740	1q42.1	Exon	C/T	0.471	0.813	0.37
*GSTP1*, rs1695	11q13	Exon	A/G	0.178	0.213	0.64
*GSTT1*	22q11.23					
*GSTM1*	1p13.3					

Notes: MAF, minor allele frequency; HWE, Hardy Weinberg Equilibrium.

* p-value for Hardy-Weinberg Equilibrium.

### Gene-gene interactions in childhood asthma

MDR was used to analyze gene-gene interaction models in childhood asthma. The two- to ten-way gene-gene interaction models are listed in Table 3. The SNP (rs1805010) in the *IL4Ra* gene had the highest testing-balanced accuracy among the 17 SNPs. A three-way interaction found between *GSTP1*, *IL4Ra* and *INSIG2* showed the highest testing-balanced accuracy and cross-validation consistency. A two-way interaction model of *IL4Ra* and *INSIG2* also exhibited high testing-balanced accuracy and cross-validation consistency, but the testing-balanced accuracy was lower than the three-way interaction model. In order to elucidate potential two- and three-way gene-gene interactions in childhood asthma, the top ten two-way and three-way interaction models were listed (Table 4, Table 5). The rank was determined by the training-balanced accuracy of MDR. In the two-way gene-gene interaction models (Table 4), interaction between *IL4Ra* and *INSIG2* has the highest training-balanced accuracy at 56.82%. *IL4Ra* also has a statistically significant interaction with *EPHX1* exon4 in childhood asthma. For the three-way interaction models (Table 5), interaction between *GSTP1*, *IL4Ra*, and *INSIG2* had the highest training-balanced accuracy. The information gain derived by the entropy-based analysis in the MDR software package was all positive in each pair-wise combination of *GSTP1*, *IL4Ra* and *INSIG2*.

**Table 3:** Summary of MDR gene-gene interaction results.

Model	Training Bal. Acc. (%)	Testing Bal. Acc. (%)	Cross-validation Consistency
*IL4Ra*	53.98	49.04	7/10
*IL4Ra*, *INSIG2*	56.83	55.78	9/10
*GSTP1*, *IL4Ra*, *INSIG2*	60.93	57.83	10/10
*GSTP1*, *IL4Ra*, *INSIG2*, *IL13* (rs1800925)	64.99	57.75	6/10
*GSTP1*, *IL4Ra*, *INSIG2*, *ADRB2* (rs1042713), *EPHX1* exon 3	70.72	49.31	5/10

**Table 4:** Two-way gene-gene interactions of MDR analysis.

Rank	Model		Training Bal. Acc. (%)	Testing Bal. Acc. (%)	*P*-value*	*P*-value#
1	*IL4Ra*	*INSIG2*	56.82	55.78	0.011	0.007
2	*IL4Ra*	*EPHX1* exon 4	55.80	53.77	0.085	0.108
3	*IL4Ra*	*STAT6*	55.73	53.86	0.079	0.094
4	*IL4Ra*	*GSTT1*	55.58	55.58	0.018	0.010
5	*IL4Ra*	*IL4*	55.39	53.54	0.099	0.133
6	*GSTP1*	*IL13* (rs1800925)	55.34	54.73	0.037	0.037
7	*IL13* (rs848)	*ADRB2* (rs1042713)	55.34	53.75	0.085	0.110
8	*IL4*	*IL13* (rs1800925)	55.25	54.80	0.035	0.036
9	*EPHX1* exon 4	*IL13* (rs20541)	55.17	54.80	0.035	0.036
10	*IL4Ra*	*GSTP1*	55.00	53.61	0.093	0.127

* Two- or Three-way interactions were validated based on 1000 permutations.

# Two- or Three-way interactions were validated based on 1000 explicit tests.

**Table 5:** Three-way gene-gene interactions of MDR analysis.

Rank	Model			Training Bal. Acc. (%)	Testing Bal. Acc. (%)	*P*-value*	*P*-value#
1	*GSTP1*	*IL4Ra*	*INSIG2*	60.93	57.83	0.002	0.001
2	*STAT6*	*IL4Ra*	*INSIG2*	59.03	51.84	0.251	0.310
3	*IL13* (rs848)	*STAT6*	*ADRB2* (rs1042713)	59.01	55.57	0.017	0.016
4	*ADRB2* (rs1042714)	*IL4Ra*	*INSIG2*	58.82	55.31	0.023	0.022
5	*ADRB3*	*IL4Ra*	*INSIG2*	58.77	56.34	0.008	0.006
6	*IL13* (rs1800925)	*IL4Ra*	*INSIG2*	58.68	53.59	0.084	0.114
7	*EPHX1* exon 3	*IL4Ra*	*INSIG2*	58.62	54.23	0.057	0.073
8	*EPHX1* exon 3	*IL4Ra*	*IL4*	58.47	53.08	0.122	0.161
9	*ADRB2* (rs1042713)	*IL4Ra*	*INSIG2*	58.45	51.99	0.240	0.294
10	*GSTP1*	*IL4Ra*	*STAT6*	58.38	55.12	0.028	0.026

* Two- or Three-way interactions were validated based on 1000 permutations.

# Two- or Three-way interactions were validated based on 1000 explicit tests.

Moreover, the *IL4Ra* and *INSIG2* gene combination interacted with *GSTP1*, *STAT6*, *ADRB2*, *ADRB3*, *IL13* and *EPHX1* exon 3 to reveal a high training-balanced accuracy above 58.38% in childhood asthma.

### Gene-environment interactions in childhood asthma

MDR analysis was used to investigate probable gene-environment interactions in childhood asthma, and revealed the interaction between 17 SNPs and 9 environmental factors. Dampness was found to be the most important environmental factor affecting asthma susceptibility (Table 6). Two-way interactions showed higher testing-balanced accuracy and cross-validation consistency, indicating that two-way interaction models were the candidate gene-environment models in our population. The top ten two-way interaction models are shown in Table 7. The interaction of preterm birth and indoor dampness had the highest training-balanced accuracy at 59.09%. *IL4Ra*-BMI interaction also affected asthma susceptibility with a high training balanced accuracy. In addition, indoor dampness also interacted with many genes including *IL13*, *ADRB2*, and *STAT6*. The lowest training-balanced accuracy was 57.34%, higher than the training-balanced accuracy of 56.98% obtained when home dampness was a single predictor (Table 6). In the two-way interaction listed in Table 7, home dampness seems to be the most important environment factor.

**Table 6:** Summary of gene-environment interaction results.

Model	Training Bal. Acc. (%)	Testing Bal. Acc. (%)	Cross-validation Consistency
Dampness	56.98	56.98	10/10
Preterm birth, Dampness	59.09	55.60	8/10
*IL4Ra*, *INSIG2*, BMI	62.00	52.17	5/10
*IL4Ra*, *INSIG2*, *IL13* (rs20541) , BMI	67.88	50.60	4/10
*IL4Ra*, *INSIG2*, *EPHX1* exon 3, *ADRB2* (rs1042713), BMI	76.76	49.30	9/10

**Table 7:** Two-way gene-environment interactions of MDR analysis.

Rank	Model		Training Bal. Acc. (%)	Testing Bal. Acc. (%)	*P*-value*	*P*-value#
1	Preterm birth	Dampness	59.09	55.60	0.003	1.000
2	*IL4Ra*	BMI	58.42	55.26	0.008	1.000
3	Dampness	BMI	58.27	56.88	0.002	0.934
4	Carpet	Dampness	58.04	58.04	0.001	0.701
5	*STAT6*	Dampness	57.85	57.22	0.001	0.914
6	*IL13* (rs20541)	Dampness	57.75	56.41	0.002	0.967
7	*IL13* (rs1800925)	Dampness	57.54	57.54	0.001	0.883
8	Preterm birth	BMI	57.49	56.42	0.002	0.966
9	*in utero* ETS	Dampness	57.40	56.80	0.002	0.947
10	*ADRB2* (rs1042714)	Dampness	57.34	57.03	0.001	0.914

* Two-way gene-environment interactions were validated based on 1000 permutations.

# Two- or Three-way interactions were validated based on 1000 explicit tests.

### Validation of gene-gene and gene-environment interactions

**Figure 1:**
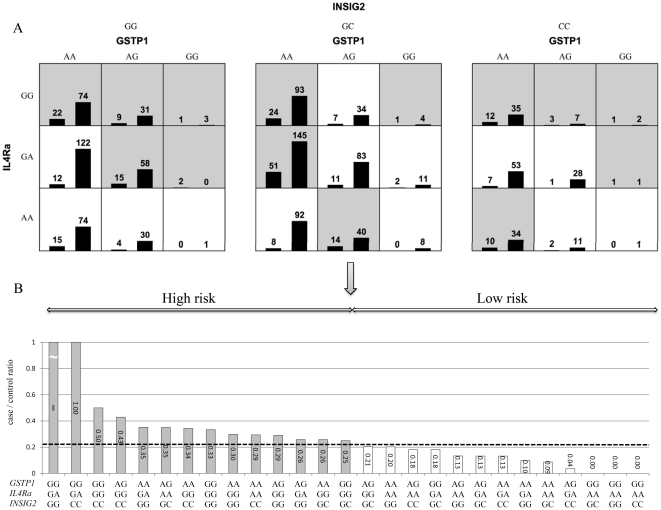
The best three-way gene-gene interaction between *IL4Ra*, *INSIG2*, and *GSTP1* for childhood asthma. (A) The 27 genotype combinations displayed by MDR. High-risk genotype combinations are in grey and low-risk genotype combinations in white. The left bar represent the asthma group and the right bar represent the controls. (B) The validation of high and low risk classification by Chi-square test.

**Figure 2:**
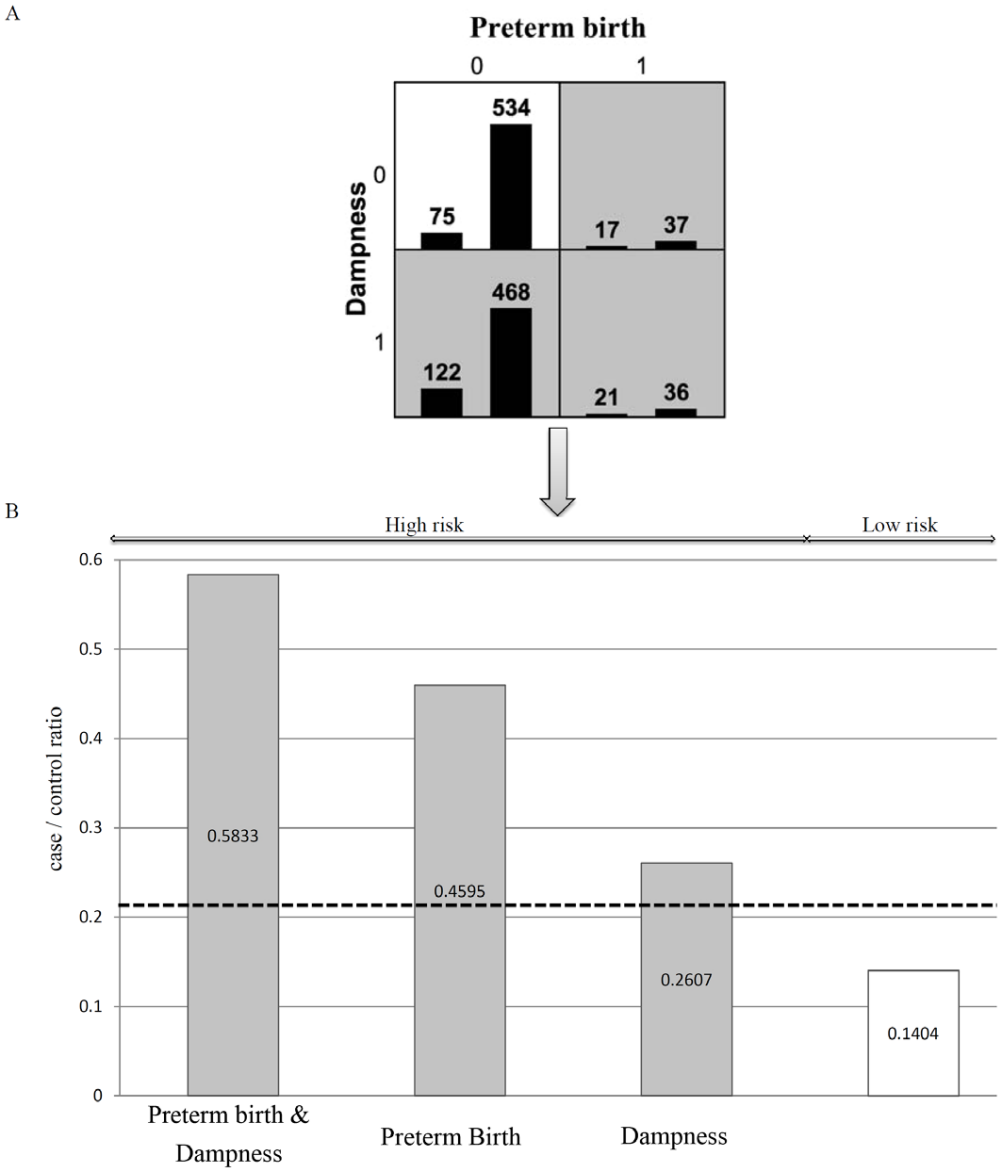
The best two-way gene-environment interaction between preterm birth and home dampness for childhood asthma. (A) The 4 gene-environment combinations displayed by MDR. High-risk genotype combinations are in grey and low-risk genotype combinations in white. The left bar represent the asthma group and the right bar represent the controls. (B) The validation of high and low risk classification by Chi-square test.

Two-way and three-way gene-gene and gene-environment interactions were examined using the detailed interaction model of MDR (Fig. 1 and Fig. 2). We used the likelihood ratio tests to validate the gene-gene interactions between *IL4Ra* and *INSIG2* on childhood asthma and the *P*-value was 0.029. The three-way interaction between *GSTP1*, *IL4Ra*, and *INSIG2* genes was also significant (P for LRT interaction = 0.003).

Chi-square tests were used to validate the high risk and low risk phenotype classification. The dimensional reduction of the three-way gene-gene interaction between *GSTP1*, *IL4Ra* and *INSIG2* is shown in Fig. 1A. Of the 27 combinations of three-way gene-gene interaction, *GSTP1* GG, *IL4Ra* GA and *INSIG2* GG resulted in the highest risk for childhood asthma. Chi-square tests also showed statistical significance (P<0.001) (Fig. 1B). The detailed model of two-way gene-environment interaction between high risk and low risk groups of childhood asthma is shown in Fig. 2A. In the two-way gene-environment interaction model shown in Fig. 2B, the interaction between preterm birth and indoor dampness revealed the highest risk at 59.09%. The lowest risk combination in preterm birth and dampness was 14.04%. Chi-square tests also showed significant differences between high risk and low risk classification on childhood asthma.

## Discussion

To the best of our knowledge, this study is the first to elucidate potential interactions between antioxidative, inflammatory and obesity-related genes on childhood asthma. Using an MDR approach, our study suggests a three-way gene-gene interaction between inflammatory gene *IL4Ra*, obesity-related gene *INSIG2*, and antioxidative gene *GSTP1*. The three-way gene-gene interaction between *IL4Ra*, *INSIG2*, and *GSTP1* was not only identified in gene-gene analyses but also confirmed in gene-environment analyses in MDR approach. The entropy-based analysis indicated that the interaction between *GSTP1*, *IL4Ra* and *INSIG2* was synergistic. The results of the gene-environment interaction analyses showed that there was an association between preterm birth and home dampness among schoolchildren. Children carrying asthma candidate genes were more susceptible to adverse effects of home dampness.

The link between asthma and obesity in childhood has been examined in many epidemiological studies [24], [25], [26]. A recent meta-analysis showed that children with high body weight were at increased risk of developing asthma [7]. Patients with allergic asthma showed significant higher BMI and insulin resistance than non-asthmatic controls [27]. Some adipokines secreted by adipose tissue have pro-inflammatory effects and also show the potential to modulate the Th2 immunity [28]. SNP rs7566605 in *INSIG2* gene is a common genetic variant associated with obesity [29]. Herbert and colleagues reported the association between SNP (rs7566605) upstream of the *INSIG2* with higher BMI [29]. The SNP (rs7566605) genotype CC was significantly associated with obesity. In animal models, the *INSIG1* and *INSIG2* gene double-knockout mice were found to be more obese than the control mice [30]. We found that the *INSIG2* gene played a key role in the three-way gene-gene interaction (Table 3). Our results constitute new evidence that obesity-related genes may show interactive effects with asthma candidate genes, such as antioxidative and Th2 pathway inflammatory genes.

Although the underlying mechanism for the link between asthma and obesity is still not fully understood, some pathophysiological pathways have been suggested , such as altered lung mechanics, enhanced systemic pro-inflammatory state, shared inherited predispositions and dietary intake [31], as well as the increased systemic oxidant stress [8], [32]. In a community-based study, Keaney et al. reported that BMI was strongly associated with the increased systemic oxidative stress, estimated by 8-epi-PGF2α [33]. Correlation between fat accumulation and systemic oxidative stress was also found in animal models. In obese mice, reactive oxygen species (ROS) level was noted to be increased selectively in adipose tissue, accompanied by decreased expression of antioxidative enzymes [34]. ROS is responsible for many chronic lung diseases such as asthma, and is proposed to be the major source of cell and tissue damage [4]. Glutathione-transferases (GSTs) play important roles in airway antioxidant defenses [4], and the*GSTP1* gene contributes more than 90% of GST-derived enzyme activity in human lung epithelium [35]. Our findings suggest that the *GSTP1* gene may be the most important gene in the antioxidative gene group (Table 3). The three-way gene-gene interaction involves *GSTP1* and *INSIG2* characterize the obesity affects on antioxidative gene further influence asthma.

Obesity-associated low-grade systemic inflammation has been suggested to be a major factor mediating the asthma susceptibility in many studies [36], [37], [38]. Polymorphisms in the inflammatory gene *IL4Ra* are associated with numerous atopic diseases such as asthma [39], [40]. In a previous study among German children, the combination of the *IL4*, *IL13*, *STAT6* and *IL4Ra* genes was revealed to increase the risk of bronchial asthma up to 16.8 times compared with the effects of individual gene polymorphisms [41]. IL13 and IL4 cytokines produced by Th2 cells and inducing IgE after allergen exposure are noted to share a common receptor IL4Ra [42]. The *IL4Ra* is a key component in the induction of the Th2 lymphocyte phenotype and its antagonist improved respiratory function and asthma control in human studies [43], [44]. Results from the top ten gene-gene interaction models elucidated the *IL4Ra* gene as a hub for gene-gene interactions on childhood asthma (Table 5), supporting that the *IL4Ra* gene may be the key regulatory element of the Th2 immune response. Gene-gene interaction between *IL13* and *IL4Ra* was reported to affect asthma in white Dutch and Chinese populations [45]. Since MDR determines one optimal interaction model that can successfully predict high/low risk asthma phenotype, the combination of *IL13* and *IL4Ra* gene may not be the optimal model in our analysis.

Asthma is a complex disease affected by many genetic factors, which in turn may be influenced by environmental exposures. Taiwan is located in a subtropical climate zone, with high temperatures and humidity (monthly mean 68–80%). Home dampness is a common problem and an important environmental factor for asthma [46], [47], [48], [49]. However, few studies are concerned with the genetic modification effects of home dampness on childhood asthma [50]. In our data, the gene-environmental MDR analyses showed that home dampness is the most important environmental factor on childhood asthma (Table 7). Using home dampness as a predictor, the testing accuracy on asthma is 56.98% (Table 6). Home dampness combined with each one of the genes *STAT6*, *IL13* and *ADRB2* raised the testing accuracy on asthma higher than 57.34% (Table 7). Our findings from interaction models with significant permutated p-values but non-significant explicit p-values might be due to the strong marginal effects from environment variables.

The six-way interaction model also showed 10/10 cross-validation consistency and high training-balanced accuracy (tables 3), but this result was not followed-up due to low testing-balanced accuracy. Low testing-balanced accuracy may be caused by the model over-fitting problem, in which the generated model fits the training data too well, increasing the high generalization error [51]. The presence of noise or paucity of representative samples in the training dataset is possible causes of model over-fitting. A model that is too complex may fit the noise, leading to lower testing-balanced efficiency. In our analysis, we used a 10-fold cross-validation approach to avoid the model over-fitting problem.

Our study has some limitations. Asthma assessment was based on parental questionnaire reports. Although misclassification of asthma status may have arisen, questionnaires are widely used to define respiratory outcomes in epidemiologic studies among children [52], [53]. Another possible limitation is the retrospective recall of environmental exposures by questionnaire, which is likely to have resulted in some misclassification. However, the reliability and validity of questionnaire on measuring dampness exposure has been verified by a strong association between inspectors and self-reported dampness [51], [52]. Due to limitations of the MDR model approach, any participants with missing data were eliminated in our study, which made selection bias possible. However, in our analysis it was found that participants without missing data did not differ greatly from all eligible participants on most of the demographic factors (data not shown).

In conclusion, our study suggests that gene-gene interactions may occur between different pathophysiological pathways and a significant three-way gene-gene interaction between *GSTP1*, *INSIG2* and *IL4Ra* on childhood asthma. Home dampness combined with each one of the genes *STAT6*, *IL13* and *ADRB2* could also raise the asthma risk. Further classifying asthma into different phenotypes and whole genome association genotyping will improve the understanding of gene-gene interactions. The MDR interaction model may work as a phenotype predictor based on the genetic information to improve the clinical diagnosis.

## Materials and Methods

### Ethics Statement

The study protocol was approved by the institutional review board of National Taiwan University Hospital and complied with the principles outlined in the Helsinki Declaration. All participants gave written informed consent.

### Study population

The study protocol was approved by the institutional review board of National Taiwan University Hospital and complied with the principles outlined in the Helsinki Declaration. TCHS recruited 4,134 seventh-grade children from 14 diverse communities in Taiwan. The design and study protocol for the TCHS has been published previously [50], [54]. Information on childhood exposure to indoor dampness and health status were collected with a questionnaire, which was completed by the child's parents or guardians. In this study, a total of 3,810 children who provided their oral mucosa were subjected for genotyping. All participating children were arranged to measure height/weight and complete pulmonary function tests with functional vital capacity (FVC), forced expiratory volume in 1 second (FEV_1_) and maximal mid-expiratory flow (MMEF) recorded. The sex-specific percentage predicted pulmonary function indices were estimated by using linear regression models [55], [56].

### Definition of asthma

Children were considered to have asthma if there was an affirmative answer to the question “Has a doctor ever diagnosed this child as having asthma?” There were 235 asthmatic children in our cohort. Family history of asthma or atopic diseases, and a personal history of wheeze or bronchitis may affect asthma susceptibility. With a proper consideration for non-asthmatic selection, the control group in present study comprised of 1,075 children without wheeze, bronchitis, or family history of either asthma or atopic disease.

### Environmental exposure assessment

Environmental factors comprised *in utero* exposure to maternal smoking (*in utero* ETS), preterm birth, pet ownership, cockroaches in the home, household carpet use and environmental tobacco smoke (ETS) at home. Body mass index (BMI) was calculated as weight/(height)^2^[kg/m^2^] and we categorized the study participants into quartiles. The baseline questionnaire collected information on several dampness indices at home. Home dampness was established by an affirmative answer to one of following questions: “Have you had visible mould in the walls or bathroom in your house in the past 12 months?”; “Have you perceived mould odor in the house during the past 12 months?” and “Have you perceived wet stamps due to moisture in the ceilings, floors or walls of your house during the past 12 months?”.

### DNA collection and genotyping

Genomic DNA was isolated from cotton swabs containing oral mucosa using the phenol/chloroform extraction method [50], [54]. All oral mucosa samples were stored at −80°C. The 17 single nucleotide polymorphisms (SNPs) were assessed by real-time polymerase chain reaction (PCR) using the TaqMan Allelic Discrimination (AD) assay on an ABI PRISMTM 7900 Sequence Detector (Applied Biosystems, Foster City, CA).

### Multifactor dimensionality reduction (MDR) analysis

The selected candidate genes were classified into three categories: inflammatory genes, obesity-related genes, and antioxidative genes. Gene-gene and gene-environment interactions were detected by an open-source MDR software package [57], [58]. MDR is a model-free and non-parametrical approach method that can identify high dimensional gene-gene or gene-environment interactions in a small population [57]. The combinations of factors which provide the most information in high risk and low risk group classification are suggested to be the most significant gene-gene or gene-environment interactions. There are no underlying assumptions about the independence or biological relevance of SNPs or any other factor. Previous studies have shown MDR to be a useful method for identifying gene-gene interactions in high dimensional data [58].

The MDR algorithm determines one optimal model that can successfully predict a high risk and low risk phenotype in a study population. Subjects with missing values in any of the factors should be deleted prior to data import. Firstly, the sample dataset is divided into training and an independent testing sub dataset for cross-validation. Cross-validation aims to avoid the model over fitting problem. Secondly, an exhaustive search of a listed of *n* genetic and environmental factors is performed. For example, for two loci with three genotypes each, there are nine possible combinations. Then, the case/control ratio for each combination is counted. Finally, each combination is assigned as high risk or low risk based on the comparison to the sample population case/control ratio. If the case/control ratio of a multifactor combination is higher than the original population, then this combination is labeled as a high risk group and vice versa. Multidimensional data are reduced to one dimension with two classes via this rocess.

An MDR model with the best testing-balanced accuracy and cross-validation consistency is selected. For example, in a 10-fold cross-validation, the original dataset is divided into 10 subsets. The maximum value of cross-validation consistency is 10 if the same combination of factors is identified across all 10 subsets, and the minimum value is 1. When the number of cases and controls are not equal, balanced-accuracy weighs the classification accuracy of the two classes equally, which is more powerful than using accuracy alone. Testing balanced-accuracy is obtained from the sum of true positive plus true negative divided by the total number of samples in the testing dataset.

For those interaction models that showed higher testing-balanced accuracy, we further used permutation tests, explicit tests [59], and the likelihood ratio test (LRT) comparing a full model that included an interaction term with a reduce model to validate the MDR interaction results on childhood asthma. The significant difference between high and low risk group on childhood asthma was also validated by chi-square tests. Furthermore, we used the entropy-based analysis included in the MDR software package to determine whether the interactions are synergistic or non-synergistic [60].
